# WeChat in China’s mobile health: a bibliometric analysis of trends, hotspots, and academic contributions

**DOI:** 10.1093/jamiaopen/ooag069

**Published:** 2026-05-05

**Authors:** Rui Li, Yin Xie, Tong Wu

**Affiliations:** Emergency Department, Tongji Hospital, Tongji Medical College, Huazhong University of Science and Technology, Wuhan, 430030, China; Department of Obstetrics, Maternity and Child Health Care Hospital of Hubei Province, Wuhan, 430070, China; Key Laboratory of Cancer Invasion and Metastasis, Ministry of Education, Tongji Hospital, Tongji Medical College, Huazhong University of Science and Technology, Wuhan, 430030, China; Department of Obstetrics and Gynecology, Tongji Hospital, Tongji Medical College, Huazhong University of Science and Technology, Wuhan, 430030, China; National Clinical Research Center for Obstetrical and Gynecological Diseases, Tongji Hospital, Tongji Medical College, Huazhong University of Science and Technology, Wuhan, 430030, China

**Keywords:** WeChat, mobile health, mHealth, bibliometric, applet, official account

## Abstract

**Objectives:**

To use bibliometric methods to deeply analyze the application of WeChat in China’s mHealth field, outline its research panorama, and clarify research frontiers.

**Materials and Methods:**

Literature from January 2011 to December 2024 was sourced from the Web of Science Core Collection, and PubMed databases. The work has been registered in PROSPERO (ID: CRD420251060517). Bibliometrix, Biblioshiny, VOSviewer, GraphPad Prism, and Adobe Photoshop were used for bibliometric analysis and visualization of the included literature.

**Results:**

A total of 1633 publications were initially retrieved, and 379 eligible documents were finally included after rigorous screening based on inclusion and exclusion criteria (1254 excluded). The annual publication output showed a sharp growth from 2016 to 2021 and entered a stable phase thereafter, while the citation count has maintained a steady upward trend since 2020. China was identified as the core contributing country in this research field. Among institutional contributors, Fujian Medical University ranked first in publication output, and Sun Yat-sen University exhibited outstanding citation impact. Keyword co-occurrence analysis identified two distinct research clusters, with one focusing on clinical practice and disease-oriented research, and the other centering on research scientificity and population health research.

**Discussion:**

It reveals a growing and interdisciplinary research area, and the trends in publication outputs, citations, and research hotspots can serve as a guide for future research.

**Conclusion:**

This research thoroughly outlined the current state of WeChat in China’s mHealth, addressed research gaps, and suggested future research directions to encourage innovative applications.

## Introduction

With aging populations, there is a rise in chronic illnesses such as cardiovascular diseases, diabetes, and neurodegenerative disorders, placing a heavy burden on healthcare systems worldwide.^1^ On this context, numerous digital technologies have been to developed to enhance healthcare delivery and patient outcomes. Among these cutting-edge approaches, mobile health (also known as mHealth) has been gaining increasing traction. mHealth involves utilizing mobile communication methods to provide users with convenient and efficient medical and health services. Common mHealth devices include smartphones, tablets, wearable devices, and handheld devices. They have been used for disease prevention, diagnosis, treatment, rehabilitation, and health management.^2^ In 2020, the COVID-19 pandemic greatly speeds up the evolution of mHealth in providing healthcare solutions.^3^ Globally, mHealth has become a common practice in both high-income and low-to middle-income countries.^4^ mHealth interventions can be aligned with the cultural norms, values, and preferences of the target population to enhance their adaptability and effectiveness.^5^ As a whole, mHealth breaks the limitations of time and space, enables wider coverage of medical resources, and forms an important pillar in the medical industry.^6^

Against this global backdrop, China, as the world’s largest mobile internet market, has also witnessed rapid advancement of mHealth technologies. China has nurtured highly integrated social and service platforms that naturally overlap with healthcare demands.^7^ Among these digital platforms, WeChat stands out as the most influential and universally accessible tool. WeChat has more than 1.3 billion monthly active users,^8^ and nearly 90% of people use WeChat every day.^9^ These unique advantages enable WeChat to conveniently push health information, provide online consultation services, and carry out health management projects. For instance, using WeChat for follow-up with discharged patients showed time-saving and cost efficiency compared to traditional methods.^10^ Furthermore, incentives such as WeChat red packets could significantly increase public attention and engagement.^11^ In addition, medical services, including appointment registration, payment, and test report, can be incorporated in the WeChat electronic health card, which greatly optimizes patients’ medical experience.^12^

Despite the remarkable achievements of WeChat in China’s mHealth field, knowledge gap remains in current research. Previous studies mostly focused on the practical experiences, while comprehensive investigations based on authoritative academic databases and rigorous bibliometric evaluations remain scarce. There is a clear need for holistic, data-driven studies to clarify the developmental trajectory and structural characteristics of WeChat‑enabled mHealth. Additionally, the academic landscape of WeChat’s potential concerning mHealth from a bibliometric perspective is still lacking. With the rapid development and continuous functional upgrading of WeChat in recent years, it is urgent to conduct systematic and up‑to‑date research to match the evolving practical landscape.

We aim to conduct a rigorous, standardized bibliometric analysis to systematically map the publication trends, research hotspots, institutional contributions, and knowledge structure of WeChat applications in the mHealth field. This study helps identify effective WeChat‑based intervention models and application scenarios, as well as reveals emerging trends and unaddressed needs. By clarifying the development trajectory and key knowledge gaps, this study supports the translation of research into sustainable, scalable, and patient‑friendly mobile health practices.

## Literature review

WeChat has developed a wide range of clinical application scenarios in mHealth, relying on its large user base and instant social ecosystem. WeChat initially developed its services through official accounts, focusing on optimizing hospital workflows such as appointment scheduling, online payment, and laboratory report inquiries.^13^^,^^14^ With the widespread adoption of applet, WeChat Pay, and online medical insurance settlements, the boundaries of healthcare services have expanded to encompass remote consultations, chronic disease follow-ups, and medication delivery.^15^^,^^16^ It significantly enhances the accessibility of medical services and patient compliance. During the pandemic, WeChat played a pivotal supporting role in health code management, nucleic acid testing, and vaccine appointment scheduling.^17^ Community WeChat groups are implemented to facilitate grassroots-level information dissemination and response mechanisms, especially for nucleic acid testing information and vaccine-related information.^18^ In recent years, WeChat has further provided AI-based health consultation, intelligent interpretation of physical examination reports, and family health record management.^12^^,^^19^ These technological applications not only enhance users’ ability to access health information but also improves their self-management capabilities in health care.

A rich research system on WeChat in the mHealth field has been formed worldwide already. Studies in China mainly focus on the verification of clinical effects, user behavior mechanisms, and technology integration models. A large number of randomized controlled trials confirmed that WeChat can effectively improve chronic disease control outcomes, enhance patients’ self-management capabilities, and improve quality of life.^20^^,^^21^ Some studies have also analyzed the intention and continuous usage behavior of WeChat users in adopting mHealth services.^22^ International research pays more attention to the its application among overseas Chinese communities, cross-border telemedicine practices, and comparative analyses with international social platforms such as Facebook and WhatsApp.^23^^,^^24^ Its exemplary value in a low-cost, high-coverage mobile health model has been widely recognized. These findings provide important references for the standardized development of social platform-based mHealth worldwide.

As an analytical tool, bibliometrics is widely utilized to evaluate academic productivity and influence. Traditional bibliometric studies predominantly focus on basic static statistical metrics such as document quantity, journal impact factors, and citation distributions.^25^ The modern bibliometrics can analyze citation characteristics, international collaborations, author partnerships, and geographic distribution, thereby providing valuable references for newcomers to the field.^26^ In the era of big data, by integrating automated tools and text mining technologies, researchers can effectively reduce, filter, and validate large volumes of literature, thereby enhancing the efficiency of systematic reviews.^26^ Bibliometrics effectively compensates for the shortcomings of traditional qualitative reviews, which are susceptible to subjective judgment and fail to comprehensively reflect the full picture of a research field. It provides reliable methodological support for researchers to quickly grasp the developmental laws of a field and identify key research directions.

## Methods

### Database selection

This research followed the sequential guidelines for bibliometric analysis,^27^ and the reporting standards for bibliometric reviews.^28^ The work has been registered in PROSPERO (ID: CRD420251060517). The Web of Science Core Collection (WOSCC) and PubMed databases were chosen due to their comprehensive coverage and renowned bibliometric research capabilities. WOSCC was prioritized for its rigorous journal inclusion criteria, which ensure that only high-quality, peer-reviewed academic publications are indexed.^29^ PubMed was included as a key biomedical database, offering authoritative coverage in life sciences and the benefit of standardized MeSH terminology for precise topic retrieval. In contrast, while Scopus offers broad journal coverage in certain fields, its indexing depth and quality in specific disciplines may not match WOSCC’s standards.^30^ Similarly, Embase is highly specialized in pharmaceutical and drug-related literature. Given that data quality and consistency are more critical than sheer coverage breadth, Scopus and Embase were not included in the final search strategy.^31^

### Data collection, integration, and deduplication

To establish an effective retrieval strategy, a two-step approach was adopted. First, relevant search terms were extracted and organized from related systematic reviews and meta–analyses.^32^ Subsequently, three authors (T.W., R.L., and Y.X.) meticulously reviewed and refined these terms to ensure comprehensive coverage of all key terms relevant to the research topic. According to the collaborative evaluation, the final search strategy was formulated using Boolean logic operators: (“wechat” or “weixin” or “official account” or “public account” or “applet” or “mini program”) and (medic* OR illness* OR disease* OR health* OR pharma* OR drug* OR therap*). A similar search was conducted in the PubMed database (Table S1). Since WeChat started its public beta testing in 2011, we have limited the time period from January 2011 to December 2024. There were no language limitations during the search.

All retrieved records were first imported into EndNote X21. Duplicate records were initially identified using the built‑in automatic deduplication function, followed by a manual cross‑check to remove remaining duplicates caused by differences in author names, publication formats, or metadata entries.

### Selection of eligible studies

Two reviewers (Y.X. and R.L.) independently screened the titles and abstracts in a stepwise manner according to the predefined inclusion and exclusion criteria. Papers were included if they: (1) were original articles; (2) used WeChat in mHealth. Papers discussing the following topics were excluded: (1) were clinical trial protocol; (2) just reveal the relationship between the use of WeChat and other induces, such as mental health; (3) Computational analysis, such as content analysis, framework development; (4) used WeChat for questionnaire assignment and collection; (5) investigate the attitude toward WeChat; (6) irrelevant with WeChat. Study designs (eg, observational, experimental) were not restricted in the selection process. Any discrepancies between the two reviewers during the screening process were resolved through discussion until consensus was reached. If no agreement could be achieved, the senior author (T.W.) was consulted to make the final decision. After selection, all eligible records were compiled into a single dataset for further screening. To facilitate subsequent bibliometric analysis and ensure data consistency, the literature formats of records retrieved from PubMed were standardized and adjusted to align with the format of WOS records. The complete process of literature retrieval and selection is summarized in Figure S1.

### Data pre-processing and extraction

Prior to bibliometric analysis, systematic data cleaning and standardization were performed using the bibliometrix and biblioshiny R packages.^33^^,^^34^ First, standardization was performed for core bibliographic entities. Author names were standardized to resolve inconsistencies in abbreviations, initials, and name order; for example, variants such as “Li, M.,” “Li, Ming,” and “Ming Li” were unified to a consistent format. Institutions, countries, and journal names were also standardized to correct spelling errors, formatting discrepancies, and aliases. Next, keyword standardization was conducted to eliminate terminological heterogeneity. Synonyms, aliases, and singular/plural forms were merged to ensure analytical consistency and research quality; for instance, terms such as “WeChat-mHealth,” “mHealth on WeChat,” and “WeChat-based mHealth” were consolidated into a unified term. Subsequently, key bibliographic fields, including first author, corresponding author, country, organization, and keywords were harmonized to ensure data completeness and consistency across all records. Finally, the cleaned bibliographic records were transformed into a structured data frame, which was suitable for subsequent bibliometric analysis.

### Bibliometric and visualization techniques

A hybrid bibliometric framework integrating multiple specialized software platforms was used to conduct a systematic, multi-dimensional exploration of the included literature.^35^ Biblioshiny was used to perform descriptive statistical analyses, map country-level collaborative relationships, quantify author outputs, visualize article distributions, and conduct cluster analysis of author keywords.^34^ GraphPad Prism was applied to analyze annual publication trends, characterize publication distributions, generate heatmaps for productive universities and active journals. VOSviewer was used to construct and visualize institutional collaboration networks and keyword co-occurrence heatmaps. A Venn diagram approach was adopted to explore collaborative relationships among highly productive authors. Bradford’s law was further applied to identify core journals within the first Bradford zone, representing the most influential publication venues in the field.

VOSviewer (v1.6.20) using co-occurrence as the analytical type and author keywords as the unit of analysis, with full counting applied during network construction. A custom thesaurus file was used to standardize terminological variations. Association strength normalization was implemented, with attraction and repulsion parameters set to 6 and 1, respectively. Clustering was performed using a resolution parameter of 0.85, a minimum cluster size of 1, with automatic merging of small clusters, yielding a final network consisting of 45 items, 232 links, and a total link strength of 461. All figures were refined in Adobe Photoshop 2025. All analytical procedures were documented in full to ensure methodological transparency, reproducibility, and consistency.

### Bibliometric key metrics

The annual citation impact was calculated by dividing the total citation impact by the years since the article was published. Category normalized citation impact was adopted to evaluate citation performance relative to the JCR subject category. The H-index (with self-citations) was used to balance research quantity and influence, defined such that an author or institution with an H-index of H has published at least H papers, each cited at least H times. Cited half-life, the number of years required for a journal to accumulate 50% of its total citations, characterizing the sustained relevance of published content. Journal impact factor, the average citations received per article in the journal over the preceding two years, a standard measure of journal standing.

## Results

### Global trend in publication outputs and citations

**Figure 1:**
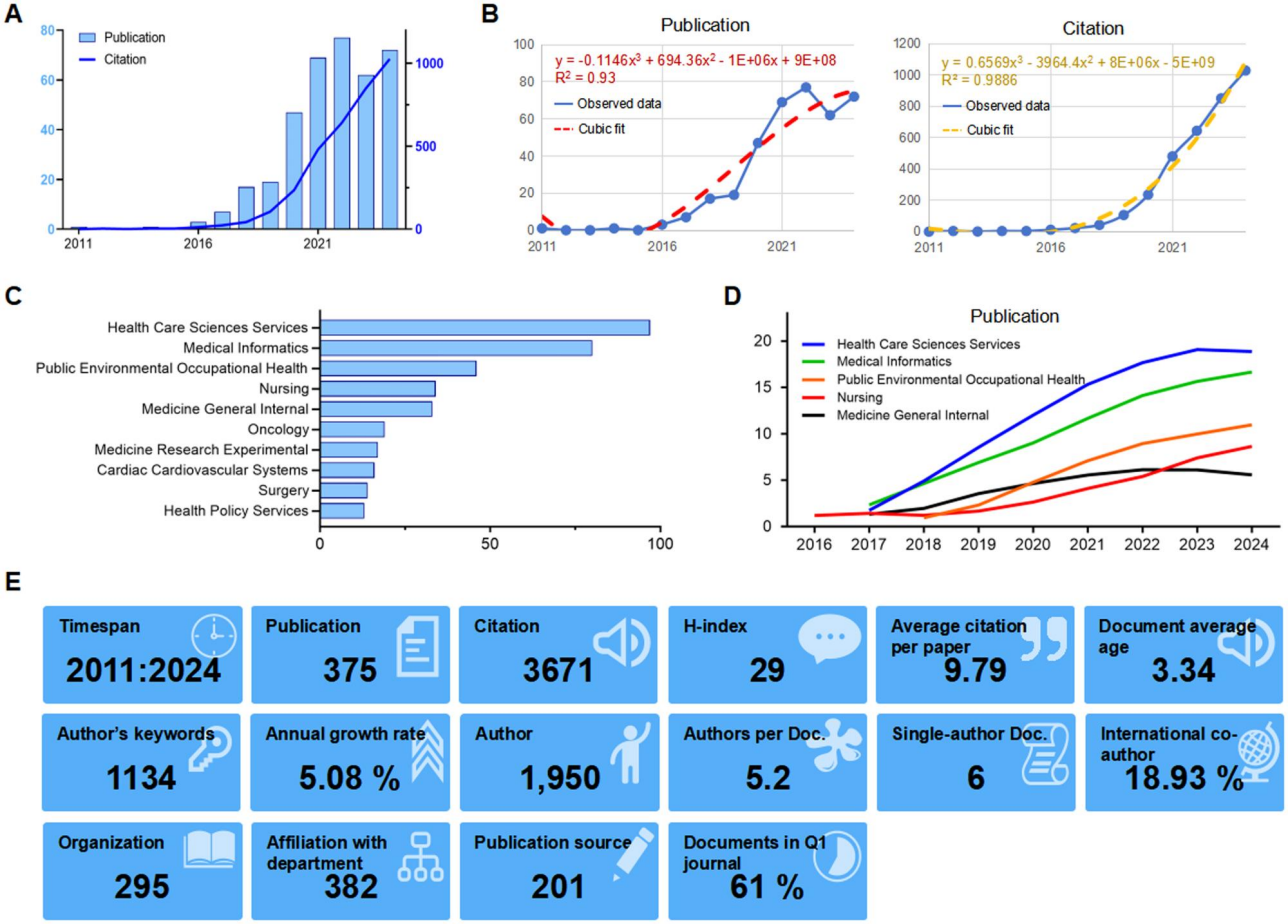
Basic bibliometric details of all relevant documents. (A) A bar and line chart depicting the annual document and citation numbers. (B) Cubic polynomial fitting of annual publications and citations. (C) Top 10 subject categories of the research. (D) Annual publication numbers for the five leading disciplines. (E) Basic bibliometric outline of the literature included.

Initially, 1633 publications were retrieved from the search (Figure S1). After excluding papers according to the inclusion and exclusion criteria (*n* = 1254), 379 documents were deemed eligible for the study. The overall publication quantity presents an S-shaped curve (Figure 1A). Before 2016, only a few articles were published each year. From 2016 to 2021, there was a sharp increase, and since 2021, it has maintained a stable trend, with around 70 articles published each year. Although the publication rate has plateaued, the number of citations has been on a linear rise since 2020, reflecting sustained attention to this field in recent years. ith informatics providing critical methodological support. Cubic polynomial fitting confirmed the accelerating growth of both publications and citations, with R^2^ values exceeding 0.90 (Figure 1B). Regarding the research areas, most documents belonged to the Health Care Sciences Services (*n* = 97, Figure 1C) and Medical Informatics (*n* = 80) categories, followed by Public Environmental Occupational Health (*n* = 46), Nursing (*n* = 34), and Medicine General Internal (*n* = 33). The number of articles on Nursing exceeded that of articles on Medicine General Internal in 2023, because most of the current projects are initiated by nurses (Figure 1D). Detailed references, journal sources, and statistical information can be found in Figure 1E.

### Academic performance of countries and institutions

**Figure 2:**
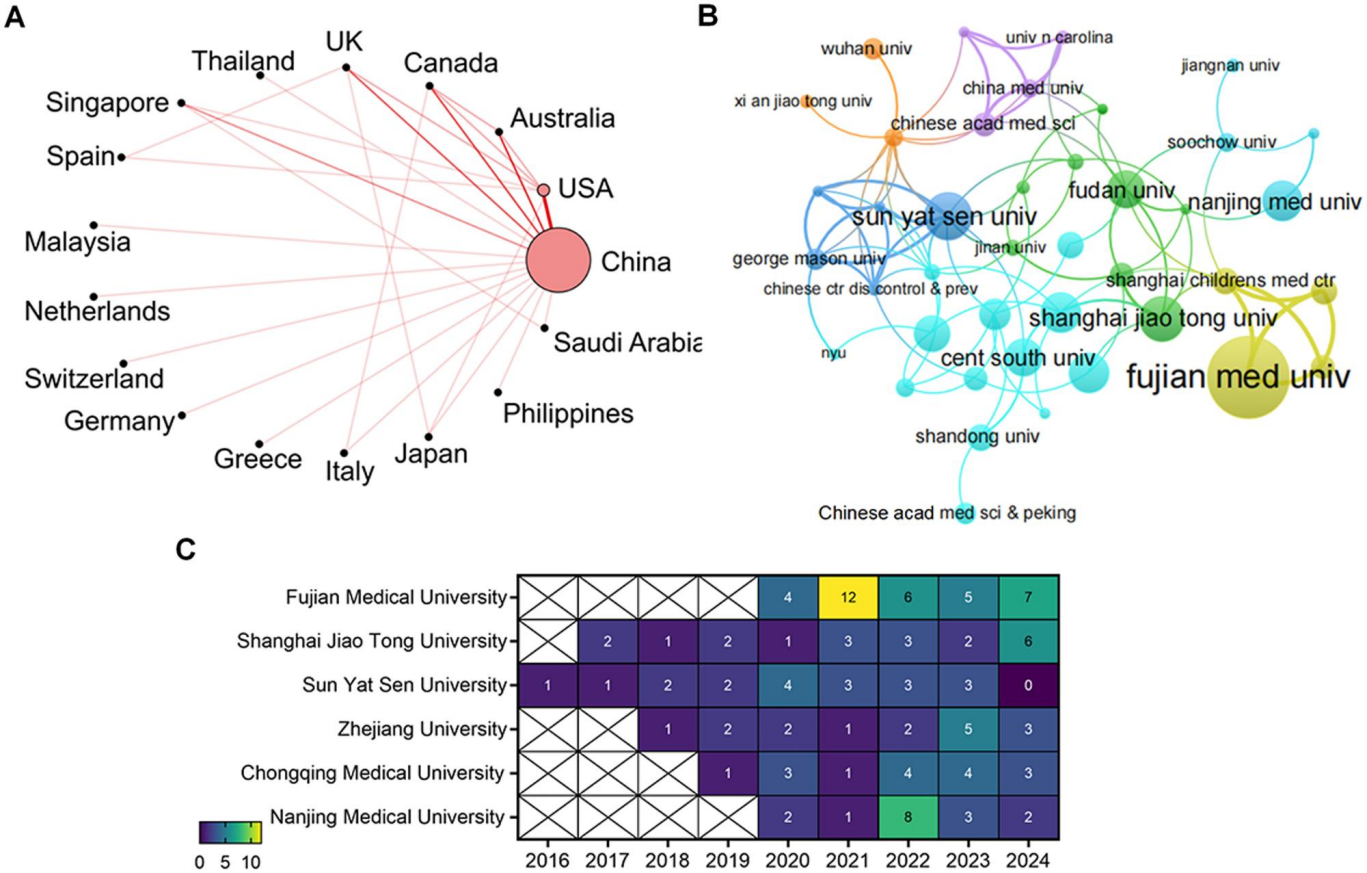
Nations and organizations engaged in the utilization of WeChat for mHealth. (A) Links between the countries involved. (B) Grouping of key institutions. (C) Yearly academic output for the top six institutions.

The examination of national and institutional publications and citations provides a comprehensive understanding of the scientific ecosystem. Seventeen countries were involved in academic exchange, and the majority of the publications were from China (Figure 2A, Table S2). Altogether 295 institutions participated in this field, and they can be divided into seven groups (Figure 2B). Fujian Medical University distinguished itself as a productive contributor (*n* = 34), while Sun Yat Sen University possessed the most citations (*n* = 386) and citation impact (*n* = 20.32). Notably, all papers of Sun Yat Sen University were published in journals indexed in Journal Citation Reports. It underscores the university’s consistent commitment to upholding high academic standards and rigorous research quality.

### Contributions of authors

**Figure 3:**
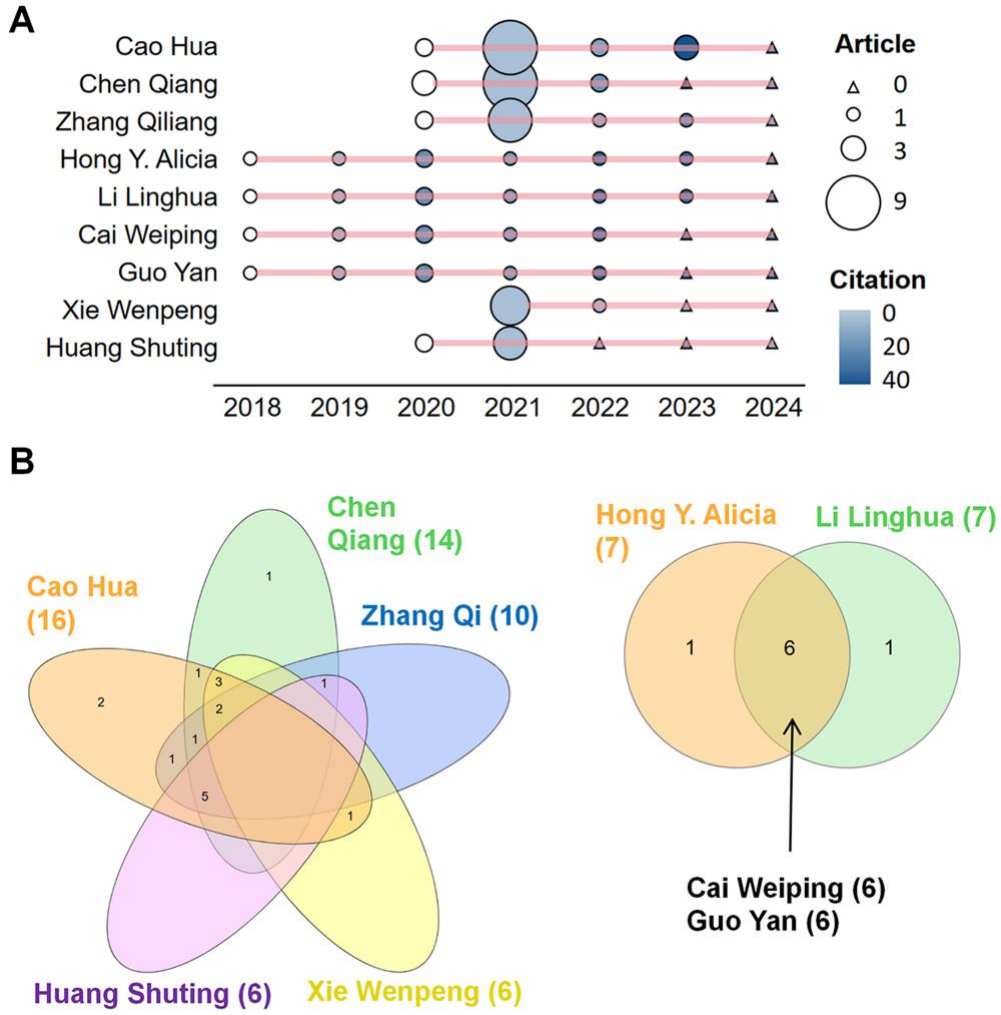
Evaluation of publication interactions. (A) Yearly volume of publications and citations for prominent authors. (B) Venn diagram showing co-authorship connections among the authors.

Among the 1950 authors involved, Cao Hua (*n* = 16), Chen Qiang (*n* = 14), and Zhang Qiliang (*n* = 10) were the top three most productive authors (Table S3), and all of them affiliated with Fujian Medical University. They received the most citations in 2021, which was exactly the second year after they first published their articles (Figure 3A). These prolific authors can be clearly divided into two groups according to the signatures on their published articles (Figure 3B). When considering global impact, Hong Y. Alicia (affiliated with Texas A&M University) and Li Linghua (affiliated with Eighth People’s Hospital) stood out in the ranking, and they jointly published six papers. It also shows the importance of publishing papers in international journals.

### Landscape of the source journals and highly cited articles

**Figure 4:**
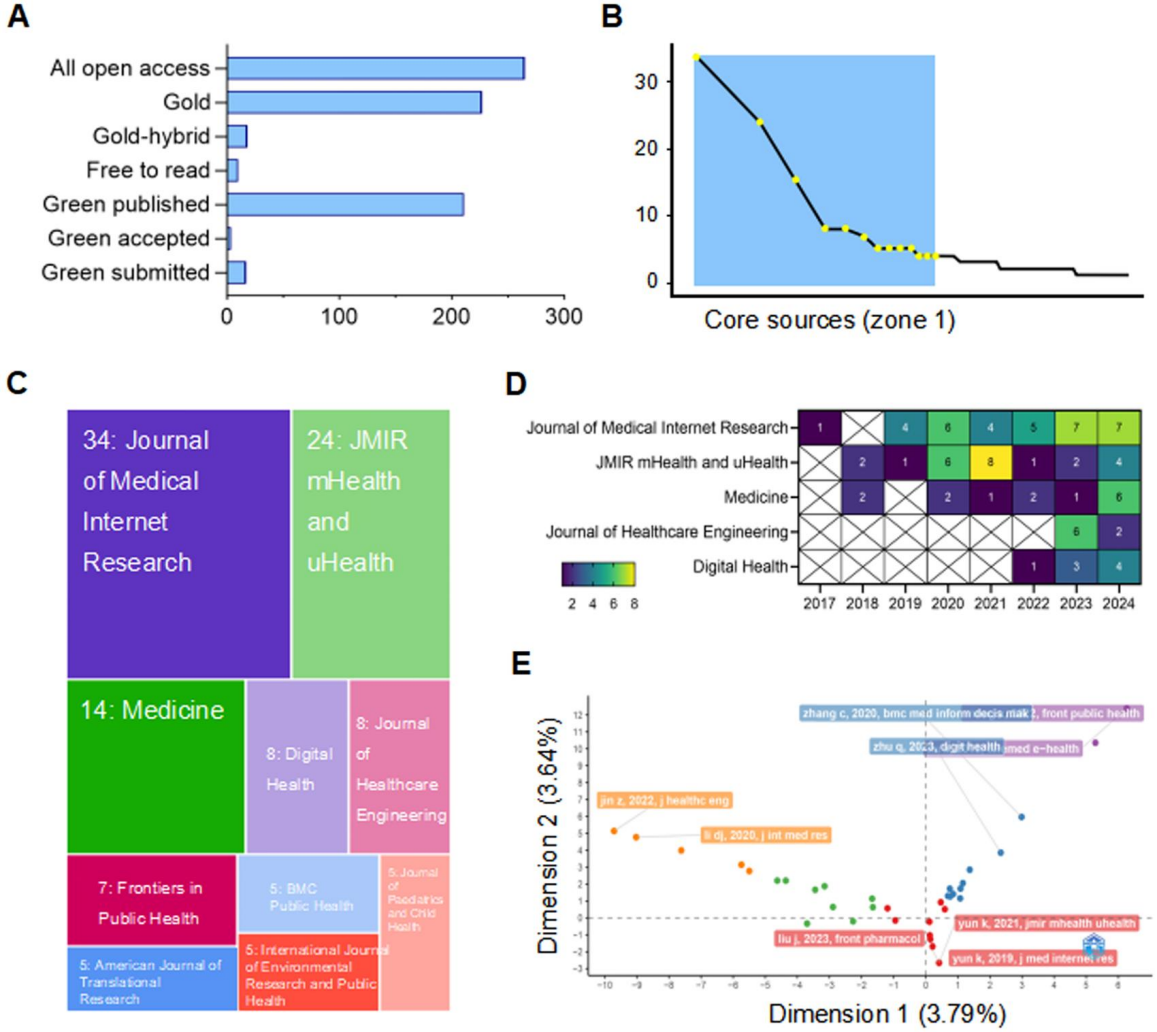
Indexes associated with journals. (A) Types of open access available for journals. (B) The first 13 journals are core as per Bradford’s law. (C) Treemap of high-active journals. (D) Annual output of publications from the top five journals. (E) PCA analysis divides relevant articles into five categories.

Out of the 201 journals examined, a large percentage (70.67%) provided open access (OA), with gold access making up 60.53% of these (Figure 4A). Furthermore, using Bradford’s law, the journals were divided into three zones, with 13 core source journals falling within zone one (Figure 4B). Journal of Medical Internet Research (*n* = 34), JMIR mHealth and uHealth (*n* = 24), and Medicine (*n* = 14) emerged as the top three most productive journals (Figure 4C and D, Table S4). The publishers were diverse, with two of the top ten journals being published by the JMIR Publication. Four of the journals fell into the Q1 quartile. It is worth noting that Journal of Medical Internet Research has been publishing papers on the application of WeChat in mHealth since 2017. This long-standing focus reflects the journal’s early recognition of WeChat’s unique potential in health care.

**Figure 5:**
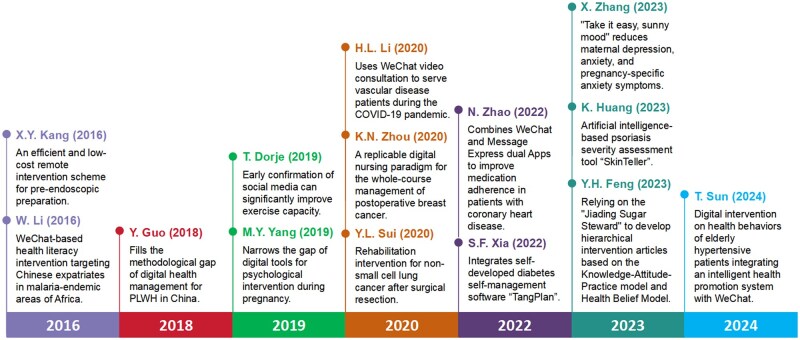
Milestone studies shaping the field of WeChat in mHealth.

Factorial map of the documents with the highest contributes suggested that these papers could be categorized into five groups (Figure 4E). As illustrated in the timeline, highly cited landmark publications collectively trace a clear evolutionary trajectory (Figure 5). From 2016 to 2018, early-stage studies focused primarily on practical, low-cost remote intervention solutions for specific clinical scenarios. From 2019 to 2020, the field expanded to broader clinical populations and chronic disease management, with trials validating WeChat’s efficacy in cardiac rehabilitation, perinatal mental health, and postoperative cancer care. Entering the 2020s, studies have integrated WeChat with artificial intelligence, cross-app digital systems, and theoretical frameworks to deliver tailored interventions. It reflects a shift from simple communication tools to comprehensive, closed-loop digital health ecosystems.

### Research hotspots and trends

**Figure 6:**
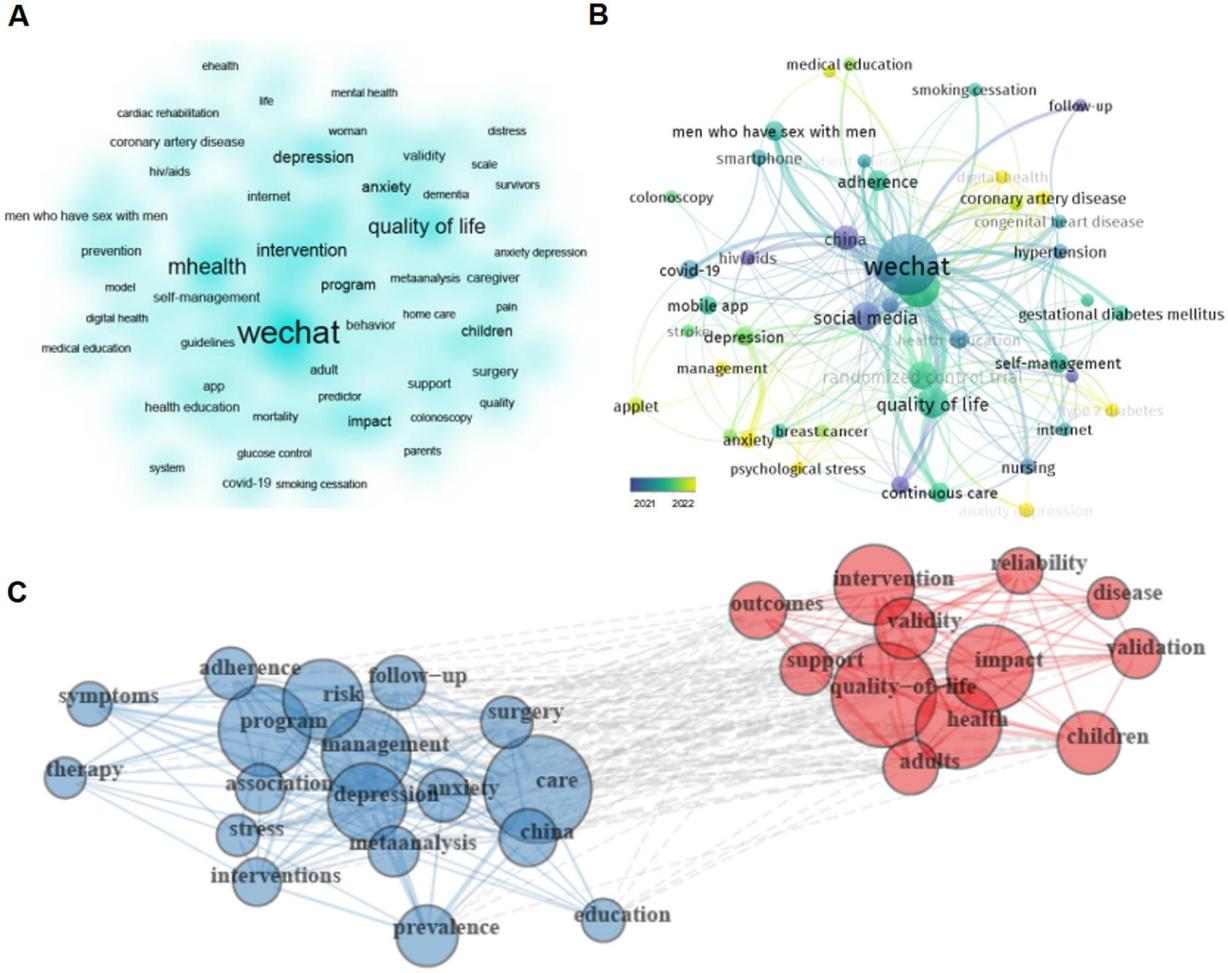
Keywords indicating the main areas and groupings. (A) Leading keywords in the mHealth sector. (B) Timeline of keywords clusters. (C) All keywords were categorized into two groups.

Keywords are essential in highlighting the main themes, areas of focus, and possible future advancements. A total of 1134 keywords were identified from the titles and abstracts of the research materials. The resulting heatmap highlighted several high-frequency keywords, including “wechat,” “quality of life,” “mHealth,” “intervention,” and “depression” (Figure 6A). Temporal analysis further revealed that the majority of these high-frequency terms reached their peak occurrence during 2021-2022 (Figure 6B). Regarding the keyword clusters, an inseparable relationship exists between the two groups (Figure 6C): the blue group is closely related to medical practice (surgery, care, management, risk, etc), and disease research (stress, therapy, symptoms, etc). While the red group has a stronger connection with research scientificity (outcomes, reliability, validation, etc.) and population health (adults, children).

## Discussion

### Bibliometric characteristics and core findings

#### Publication and citation dynamics

This study used bibliometric methods to analyze the application of WeChat in China’s mHealth field. It revealed that the number of relevant publications presented an S-shaped curve with three key timepoints: 2011, 2016, and 2021. The citation count has been rising linearly since 2020. At the national and institutional levels, China contributed the majority of the research, and Fujian Medical University had the highest number of publications. Over 70% of involved journals offered open access, and Journal of Medical Internet Research was the most productive journals. The keyword clustering reflected the hot topics in this field. Our research comprehensively depicted the research status of mHealth based on WeChat. As far as we know, it is the first time to provide a clear landscape, and fill the gap from a bibliometric perspective.

The S-shaped curve of publication quantity indicates that this field experienced a slow start before 2016, likely due to the relatively new nature of WeChat-based mHealth applications, and the time required for researchers to recognize its potential. The sharp increase from 2016 to 2021 may be attributed to the growing popularity of WeChat, the development of mHealth technology, and increasing awareness of the benefits of integrating the two in healthcare delivery. Through methods such as WeChat group management, official account recommendation and applets, WeChat can effectively promote the change of healthy behaviors and the dissemination of health information. In a study targeting dementia caregivers in China and the United States, WeChat was used as a culturally tailored intervention platform, significantly improving participants mental health.^36^ WeChat applets have been shown to improve patient compliance during Helicobacter pylori eradication therapy.^37^ In another study, patients with neurogenic bladder found the program easy to use and it effectively guides bladder management.^38^

#### Institutional and author contributions

Although the number of papers published by Sun Yat-sen University is not the largest, it has the highest number of papers published in journals indexed in Journal Citation Reports. As a result, its citation is higher than that of Fujian Medical University. This fact emphasizes the importance of high-quality publications in establishing a strong academic reputation.^39^ Similar to our idea, it is found that surgeons with higher H-index were more likely to receive NIH funding.^40^ Therefore, prioritizing the quality of research papers is more effective than merely increasing the quantity.

The international collaboration among Hong Y. Alicia, Li Linghua and other authors underscores the value of cross-border research. The pandemic of COVID-19 also emphasizes the value of these partnerships in increasing worldwide impact. In New Zealand, serological assays for SARS-CoV-2 were successfully performed during a nationwide lockdown thanks to the generous international sharing of reagents and technical expertise.^41^ The Global Obstetrics Network Initiative exemplifies that international collaboration can foster communication and cooperation among groups conducting clinical trials and observational studies, especially in optimizing resource use and minimizing duplication.^42^ Altogether, international collaboration is increasingly recognized as a vital component in addressing global health challenges and advancing scientific knowledge.

#### Source journal characteristics

OA journals are now a key element of academic publishing, especially in sectors where fast dissemination is essential. The high proportion of OA journals enhances the visibility of research and attracts a larger readership. At the level of individual papers, OA articles typically receive more citations than those that are not OA.^43^^,^^44^ Nevertheless, the related costs may restrict the creation and dissemination of knowledge, particularly for researchers in low-income nations.^45^ To overcome these challenges, efforts include programs like Pre-Publication Support Services, which offer training and editorial support to researchers from low- and middle-income nations.^46^ It helps them to navigate the publication process without incurring additional costs. Overall, the trend toward OA is regarded as a positive development for the scientific community, as it aligns with the broader goal of making knowledge freely available to everyone.^47^

#### Keyword clustering and thematic focus

The two keyword clusters are a critical theoretical marker of the maturation and iterative development of WeChat-based mHealth. The initial stage of mHealth research was characterized by exploratory practice, as researchers sought to understand the potential and limitations of these emerging technologies. This exploratory phase was marked by a focus on understanding the feasibility and effectiveness of mHealth interventions. For instance, the use of mHealth technologies in managing hypertension and hyperlipidemia demonstrated early potential due to their cost-effectiveness and ability to enhance patient engagement compared to traditional methods.^48^ It also involves the understanding of their application in low-resource settings.^49^ The emergence of keywords such as outcomes, reliability, and validation reflect that the academic community has begun to pay attention to the scientificity and normative evaluation. Researchers may use rigorous research methods (eg, randomized controlled trials, long-term follow-up) to verify the clinical efficacy.^50–52^ These studies highlight the potential of mHealth interventions to foster sustainable health behavior changes, albeit with a need for more methodologically rigorous studies to confirm these effects over extended periods. Additionally, the Health IT Usability Evaluation Model is one such framework that has been applied to evaluate the usability of mHealth technology, demonstrating its utility in identifying key usability factors such as performance speed and information needs.^53^

### Implications and future development

#### Integration of AI-driven analytics

WeChat’s massive user base and real-time data collection capabilities provide an unprecedented dataset for AI model training. Integrating predictive analytics, personalized recommendation algorithms, and intelligent diagnostic tools into WeChat applets or official accounts can make it a proactive decision-support system. For instance, the development of intelligent medication management systems on WeChat has shown to improve medication adherence and patient satisfaction significantly.^54^ The personalized recommendation algorithms within WeChat can analyze user data to provide tailored health advice and interventions, as demonstrated in shopping, where personalized recommendations have been shown to improve user satisfaction and engagement.^55^ AI-driven WeChat mHealth applications can realize personalized chronic disease management plans, early risk warning for common diseases, and intelligent interpretation of basic health data. It aligns with the growing trend of utilizing digital platforms to offer a scalable and accessible solution to meet the diverse needs of patients.^56^

#### Interoperability with hospital information systems

Improving the interoperability between WeChat platforms and hospital information systems (HIS) is essential to break down data silos. Future research should focus on developing standardized data interfaces and unified information exchange protocols to realize secure and real-time data sharing between WeChat and HIS, electronic health record (EHR) systems, and medical insurance platforms. The Fast Healthcare Interoperability Resources (FHIR) model exemplifies a robust framework for translating clinical data into standardized formats.^57^ Similarly, the Data Tags Suite model underscores the need for standardized ways of describing informed consent and data use agreements.^58^ This interoperability will enable a closed-loop healthcare service model, from online pre-consultation and appointment registration to offline diagnosis and treatment, and post-discharge online follow-up.

#### Ethical and regulatory challenges

The rapid development of WeChat mHealth services outpaces the formulation of targeted regulatory frameworks, leading to potential risks. The reference to the Australian Safety and Efficacy Register for New Interventional Procedures—Surgical (ASERNIP-S) provides a relevant perspective on these issues. ASERNIP-S discussion on consent and privacy underscores the need for transparent data practices and robust consent mechanisms to safeguard user information.^59^ Furthermore, the ethical considerations discussed in the ASERNIP-S article can be extended to the broader digital ecosystem. such as unregulated medical service provision and inaccurate health information dissemination. Furthermore, the ethical aspects, particularly in terms of justice and equity, must be considered to prevent exacerbating existing health disparities.^60^ The Digital Health Equity Framework suggests that digital health equity should be incorporated into health provider training to ensure fair access and benefit sharing.^61^ Additionally, cross-regional and cross-national regulatory coordination should be considered to adapt to the potential cross-border application of WeChat-based mHealth services for overseas Chinese communities.

## Limitation

Despite the comprehensive nature of this study, several limitations should be acknowledged. Firstly, although the WOSCC and PubMed are well-recognized databases, they may not capture all relevant literature. As WeChat is a platform predominantly used in mainland China, many high-quality Chinese-language studies published in local journals are not indexed. This language and database coverage bias may cause potential citation omissions, and further skew the objective evaluation of the overall research output and citation impact. Secondly, the study focused on the period from 2011 to 2024. Given the rapidly evolving nature of mHealth and WeChat, the results may not fully reflect the most recent trends and developments. The fast iteration of AI-integrated health services may have spawned new research directions that have not yet accumulated sufficient publications for bibliometric detection. It thus fails to reflect the latest practical applications and exploratory studies that are still in the early publication stage. Future research should continuously update and expand the time frame to capture the latest advancements. Thirdly, bibliometric methods cannot objectively evaluate the methodological quality or practical clinical value of individual studies.^62^

## Conclusion

This bibliometric study systematically delineates the developmental trajectory and knowledge landscape of WeChat-based mHealth in China from 2011 to 2024. The findings reveal that WeChat-based mHealth has evolved from an exploratory stage to a mature, steadily developing domain. As the dominant contributor, China has formed a distinct academic ecosystem in this field, with institutional outputs highlighting the pivotal role of high-quality research in shaping academic influence. The two core keyword clusters construct the core knowledge framework of the field, clarifying the dual focus of theoretical rigor and practical application in WeChat-based mHealth research.

WeChat’s unique advantages of a massive user base and integrated functional ecosystem have made it an indispensable digital tool in China’s mHealth sector. This study identifies three key directions for the future advancement of the field: the integration of artificial intelligence; the improvement of interoperability between WeChat platforms and hospital information systems; and the establishment of targeted ethical and regulatory frameworks. Additionally, enhancing international collaboration is critical to expanding the global applicability of WeChat’s mHealth model. While this study provides comprehensive insights, it is limited by potential omissions of Chinese-language literature and the inability to capture the latest research. Overall, this study offers actionable guidance for researchers, healthcare institutions, and policymakers, and lay a solid academic foundation for optimizing WeChat’s application in mHealth. It is anticipated that with technological innovation, institutional improvement, and international collaboration, WeChat-based mHealth will continue to drive the digital transformation of healthcare, and provide a valuable digital health solution for advancing global population health management.

## Supplementary Material

ooag069_Supplementary_Data

## Data Availability

The data underlying this article will be shared on reasonable request to the corresponding author.
