# Supplementary material for: WeChat in China’s mobile health: a bibliometric analysis of trends, hotspots, and academic contributions
Source: JAMIA Open. 2026 May 5;9(3):ooag069. doi: 10.1093/jamiaopen/ooag069 (PMC13143428; doi:10.1093/jamiaopen/ooag069)
Supplement: ooag069_Supplementary_Data [file ooag069_supplementary_data.zip › 2026.4.6 Supplementary Tables.docx]

### Supplementary Tables

**Supplementary Table S1** Specific search terms of databases.

| **Database** | **Search formula** | **Number** |
| --- | --- | --- |
| Web of Science Core Collection | ((TS= ("Wechat" OR "weixin" OR "official account" OR "public account" OR "applet" OR "Mini Program")) and (TS= (medic* OR illness* OR disease* OR health* OR pharma* OR drug* OR therap*))) | 1633 |
| PubMed | ("Wechat" or "weixin" or "official account" or "public account" or "applet" or "Mini Program") AND (medic* OR illness* OR disease* OR health* OR pharma* OR drug* OR therap*) | 1501 |

| **Supplementary Table S2** Top 10 organizations ranked by the counts of publications. | | | | | | |
| --- | --- | --- | --- | --- | --- | --- |
| **Organization** | **Doc.** | **Citation** | **% Docs Cited** | **Category Normalized Citation Impact** | **Documents in JIF Journals** | **H-Index with Self-Citations** |
| Fujian Medical University | 34 | 166 | 91.18 | 4.88 | 27 | 8 |
| Shanghai Jiao Tong University | 20 | 148 | 85 | 7.40 | 13 | 8 |
| Sun Yat Sen University | 19 | 386 | 94.74 | 20.32 | 19 | 9 |
| Zhejiang University | 16 | 178 | 93.75 | 11.13 | 14 | 7 |
| Chongqing Medical University | 16 | 72 | 87.5 | 4.50 | 11 | 6 |
| Nanjing Medical University | 16 | 71 | 75 | 4.44 | 13 | 6 |
| Fudan University | 15 | 178 | 93.33 | 11.87 | 10 | 6 |
| Central South University | 15 | 116 | 86.67 | 7.73 | 13 | 6 |
| Sichuan University | 14 | 92 | 71.43 | 6.57 | 9 | 5 |

| **Supplementary Table S3** Top nine most productive authors ranked by the numbers of publications. | | | | | | |
| --- | --- | --- | --- | --- | --- | --- |
| **Author** | **Doc.** | **Citation** | **Domestic Doc.** | **International Doc.** | **Category Normalized Citation Impact** | **H-Index with Self-Citations** |
| Cao Hua | 16 | 60 | 16 | 0 | 3.75 | 5 |
| Chen Qiang | 14 | 80 | 14 | 0 | 5.71 | 6 |
| Zhang Qiliang | 10 | 50 | 10 | 0 | 5 | 5 |
| Hong Y. Alicia | 7 | 148 | 1 | 6 | 21.14 | 6 |
| Li Linghua | 7 | 144 | 0 | 7 | 20.57 | 5 |
| Cai Weiping | 6 | 142 | 0 | 6 | 23.67 | 5 |
| Guo Yan | 6 | 142 | 0 | 6 | 23.67 | 5 |
| Xie Wenpeng | 6 | 30 | 6 | 0 | 5 | 3 |
| Huang Shuting | 6 | 33 | 5 | 0 | 6.6 | 4 |

| **Supplementary Table S4** Top 10 most productive journals ranked by the numbers of publications. | | | | | | |
| --- | --- | --- | --- | --- | --- | --- |
| **Journal** | **Doc.** | **Citation** | **Category Normalized Citation Impact** | **Publisher** | **Cited Half Life** | **JIF** |
| Journal of Medical Internet Research | 34 | 529 | 1.53 | JMIR Publication | 3.9 | 5.8 |
| JMIR mHealth and uHealth | 24 | 295 | 0.99 | JMIR Publication | 4.1 | 5.4 |
| Medicine | 14 | 103 | 0.64 | Lippincott Williams & Wilkins | 5.2 | 1.4 |
| Journal of Healthcare Engineering | 8 | 37 | 0.64 | Hindawi Ltd. | - | - |
| Digital Health | 8 | 10 | 0.87 | SAGE Publication | 2.5 | 2.9 |
| Frontiers in Public Health | 7 | 19 | 0.49 | Frontiers Media SA | 2.5 | 3 |
| International Journal of Environmental Research and Public Health | 5 | 73 | 0.87 | MDPI | - | - |
| BMC Public Health | 5 | 49 | 0.82 | BMC | 5.4 | 3.5 |
| American Journal of Translational Research | 5 | 38 | 0.57 | E-Century Publishing Corp. | 4.3 | 1.7 |
