# Supplementary figures and images for: WeChat in China’s mobile health: a bibliometric analysis of trends, hotspots, and academic contributions
Source: JAMIA Open. 2026 May 5;9(3):ooag069. doi: 10.1093/jamiaopen/ooag069 (PMC13143428; doi:10.1093/jamiaopen/ooag069)

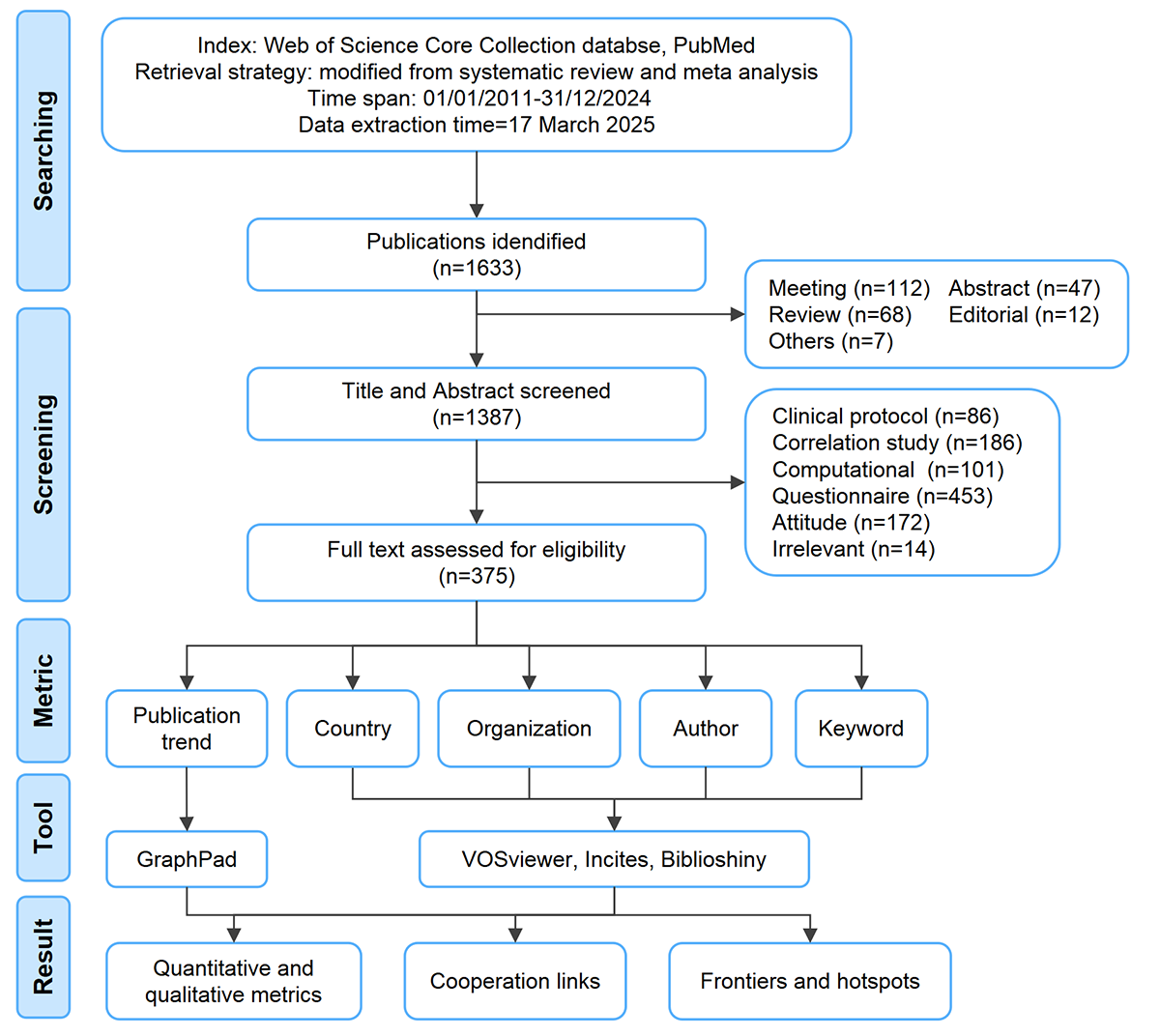

Supplement: ooag069_Supplementary_Data [file ooag069_supplementary_data.zip › Supplementary Figure 1.tif]
